# DPH1 and DPH2 variants that confer susceptibility to diphthamide deficiency syndrome in human cells and yeast models

**DOI:** 10.1242/dmm.050207

**Published:** 2023-09-22

**Authors:** Koray Ütkür, Klaus Mayer, Maliha Khan, Thirishika Manivannan, Raffael Schaffrath, Ulrich Brinkmann

**Affiliations:** ^1^ Institut für Biologie, Fachgebiet Mikrobiologie, Universität Kassel, 34132 Kassel, Germany; ^2^ Roche Pharma Research and Early Development (pRED), Large Molecule Research, Roche Innovation Center Munich, 82377 Penzberg, Germany

**Keywords:** Diphthamide synthesis, Elongation factor 2, Development, Missense, Rare disease, Intellectual disability

## Abstract

The autosomal-recessive diphthamide deficiency syndrome presents as intellectual disability with developmental abnormalities, seizures, craniofacial and additional morphological phenotypes. It is caused by reduced activity of proteins that synthesize diphthamide on human translation elongation factor 2. Diphthamide synthesis requires seven proteins (DPH1-DPH7), with clinical deficiency described for DPH1, DPH2 and DPH5. A limited set of variant alleles from syndromic patients has been functionally analyzed, but databases (gnomAD) list additional so far uncharacterized variants in human DPH1 and DPH2. Because DPH enzymes are conserved among eukaryotes, their functionality can be assessed in yeast and mammalian cells. Our experimental assessment of known and uncharacterized *DPH1* and *DPH2* missense alleles showed that six variants are tolerated despite inter-species conservation. Ten additional human *DPH1* (G113R, A114T, H132P, H132R, S136R, C137F, L138P, Y152C, S221P, H240R) and two *DPH2* (H105P, C341Y) variants showed reduced functionality and hence are deficiency-susceptibility alleles. Some variants locate close to the active enzyme center and may affect catalysis, while others may impact on enzyme activation. In sum, our study has identified functionally compromised alleles of *DPH1* and *DPH2* genes that likely cause diphthamide deficiency syndrome.

## Teaser

**Summary:** Diphthamide deficiency syndrome is caused by mutations in DPH genes. Analyses in human cell and yeast models identified functionally compromised missense alleles of human *DPH1* and *DPH2*.

## INTRODUCTION

Diphthamide deficiency syndrome is a rare autosomal-recessive disease that severely affects child development, mostly accompanied by intellectual disability (central nervous system malformations), short stature, craniofacial and additional morphological features (hand/foot anomalies), and additional clinical symptoms (Hawer et al., 2020; Shankar et al., 2022; Urreizti et al., 2020). The molecular cause of the syndrome is reduced activity of diphthamide-synthesizing enzymes, which compromises the placement of diphthamide on translation elongation factor 2 (eEF2). Thereby, overall ribosomal protein synthesis can still occur, but translation accuracy is affected at the elongation state by permitting frameshifts to occur (Hawer et al., 2018; Liu et al., 2012; Pellegrino et al., 2018). In consequence, diphthamide deficiency triggers stress responses such as hypersensitivity towards oxidative stress and NF-κB pathway pre-activation in experimental human cell models (Mayer et al., 2019; Stahl et al., 2015). The importance of diphthamide in normal development is also reflected by phenotypes of animals with inactivated diphthamide synthesis genes. Such mice show developmental phenotypes, in part reflecting craniofacial features observed in humans and embryonic lethality in complete DPH gene knockouts (Chen and Behringer, 2004; Liu et al., 2006, 2012; Shankar et al., 2022). The modification of eEF2 with diphthamide, and the pathway and enzymes for its synthesis are highly conserved in eukaryotes and archaea (Abdel-Fattah et al., 2013; Schaffrath et al., 2014). eEF2 modification by diphthamide requires the activity of seven proteins (DPH1-DPH7) and S-adenosyl methionine (SAM) as co-factor. An iron-sulfur (FeS) cluster containing DPH1/DPH2 heterodimer that needs DPH3/DPH4 for electron donation generates 3-amino-3-carboxypropyl (ACP)-modified histidine at position His715 of human eEF2. DPH5 converts the ACP intermediate to methylated diphthine, which then becomes de-methylated by DPH7 and subsequently amidated by DPH6 to result in diphthamide-modified eEF2 (Abdel-Fattah et al., 2013; Dong et al., 2014, 2019; Liu et al., 2004; Schaffrath et al., 2014; Uthman et al., 2013).

The phenotypes of diphthamide deficiency syndrome and its association with the *DPH1* gene were initially described by Loucks and colleagues as Loucks-Innes syndrome (Loucks et al., 2015). Additional patients carrying different variants were subsequently identified (Alazami et al., 2015; Nakajima et al., 2018; Riazuddin et al., 2017; Sekiguchi et al., 2018; Urreizti et al., 2020), and the effects of pathogenic DPH1 variants on enzyme functionality were experimentally proven (Urreizti et al., 2020). The phenotypes associated with compromised DPH1 function were thereafter also identified in individuals with variants that affect DPH2 functionality (Hawer et al., 2020) and recently in individuals with DPH5 variants (Shankar et al., 2022). The *DPH1* and *DPH2* alleles identified in patients are summarized in Table S1. All clinical reports so far have described the syndrome in patients that either carry defective homozygous alleles (consanguineous families) or compound heterozygous ones, with their heterozygous (DPH+/−) parents showing no phenotype. Because one functional allele for each enzyme is sufficient for activity, heterozygous defective combinations of different DPH enzymes (e.g. DPH1+/− combined with DPH2+/−) were neither observed with the syndrome, nor expected. In all cases, diphthamide synthesis of the homozygous or compound heterozygous carriers of variant alleles appeared to be reduced but not completely abolished. This agrees with the observation that complete inactivation of diphthamide synthesis causes perinatal or embryonal lethality in animals (Chen and Behringer, 2004; Liu et al., 2006, 2012; Shankar et al., 2022). So far, there are no known treatments to address this rare disease. The diagnosis of diphthamide deficiency syndrome is based upon whole-exome sequencing of patients with the above-described manifestations that identify potentially compromised DPH enzyme variants. Analyses in recombinant human MCF7 cell line derivatives or in mutated yeast cells determine thereafter whether and to what degree an identified sequence variant affects enzyme functionality (Hawer et al., 2020; Mayer et al., 2017). Human cell assays more closely reflect the disease, but the current MCF7 cell assay system is of limited sensitivity for detecting gradual activity differences. Yeast is evolutionarily rather distant to humans, but the diphthamide synthesis pathways and enzymes are highly conserved, and the yeast assay system is more sensitive at detecting gradual differences than human cell assays (Hawer et al., 2020). Thus, a combination of both is most suited to assess the functionality of potentially pathogenic human DPH variants. Diphthamide deficiency syndrome is rare, and so are the alleles that encode variants with compromised DPH functionality. So far, few rare alleles (i.e. observed in patients to define the syndrome) have been functionally characterized. Heterozygous parents with one pathogenic and one functional allele are not affected. Hence, additional variant alleles of *DPH1* and *DPH2* can be found without disease association in genetic variability databases such as gnomAD (Karczewski et al., 2020). These include alleles that encode DPH1 or DPH2 truncations or missense variants. Although most truncations can be considered inactive, functionality of most missense variants is unclear. Some alleles in databases carry labels that suggest deleterious effects proposed through bioinformatics, but, without experimental assessment, functionality or deficiency of a variant cannot be unambiguously called.

Here, we describe experimental analyses in human and yeast cell-based assays of selected DPH1 and DPH2 missense variants for which clinical phenotype and molecular functionality have not been addressed. These analyses support the interpretation of genetic analyses to diagnose diphthamide deficiency syndrome, and additionally provide further insights into the mode of action of DPH1 and DPH2 enzymes.

## RESULTS

### Identification of human *DPH1* and *DPH2* allele variants that may affect enzyme activity

**Fig. 1.**
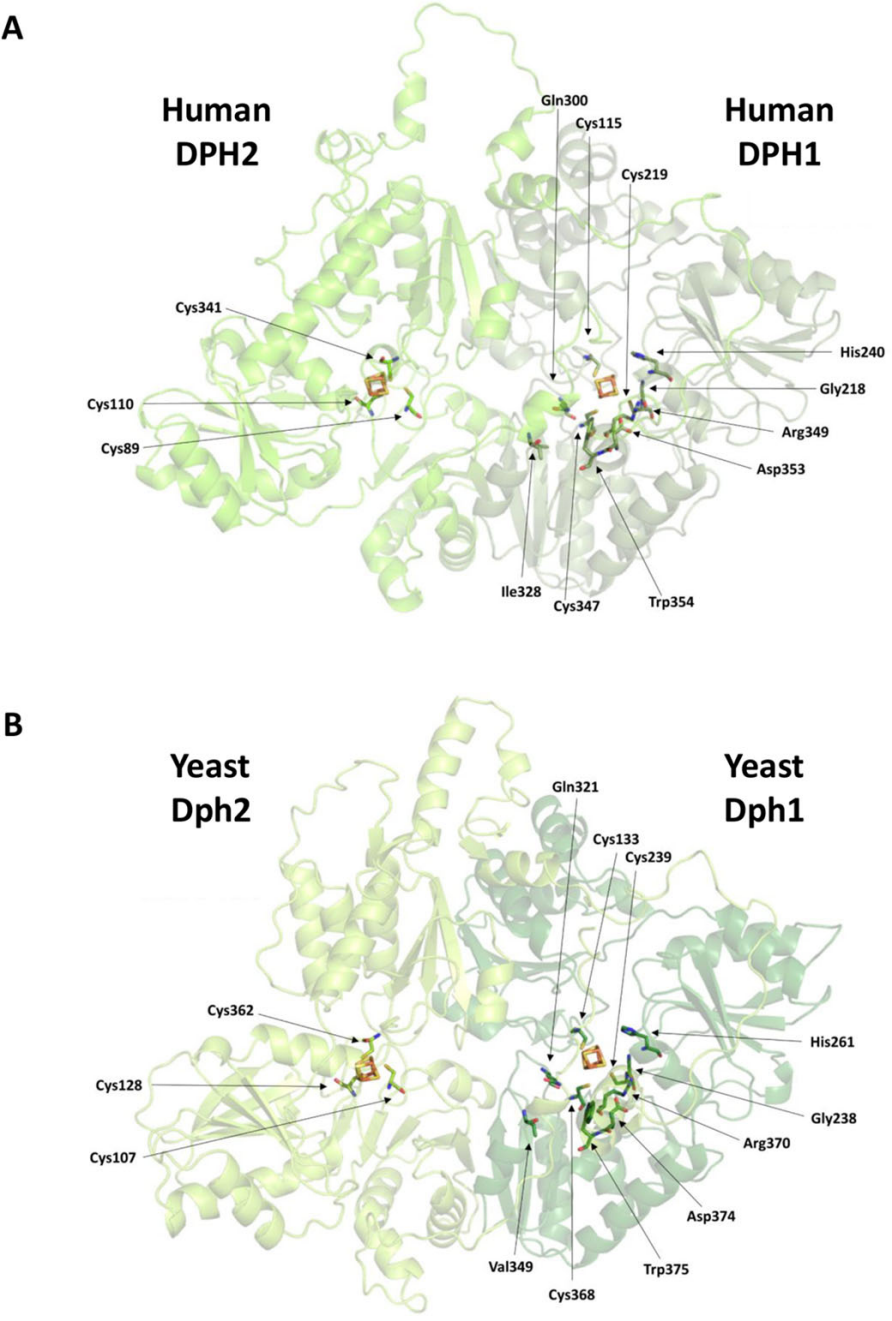
**Human DPH1/DPH2 and yeast Dph1/Dph2 structure models.** (A,B) Human DPH1/DPH2 (A) and yeast Dph1/Dph2 (B) residues that match iron-sulfur (FeS) cluster and conserved S-adenosyl methionine (SAM)-binding sites. Protein structures were modeled via the AlphaFold-based tool ColabFold (Jumper et al., 2021; Mirdita et al., 2022) and illustrated using PyMOL version 1.3.

**Fig. 2.**
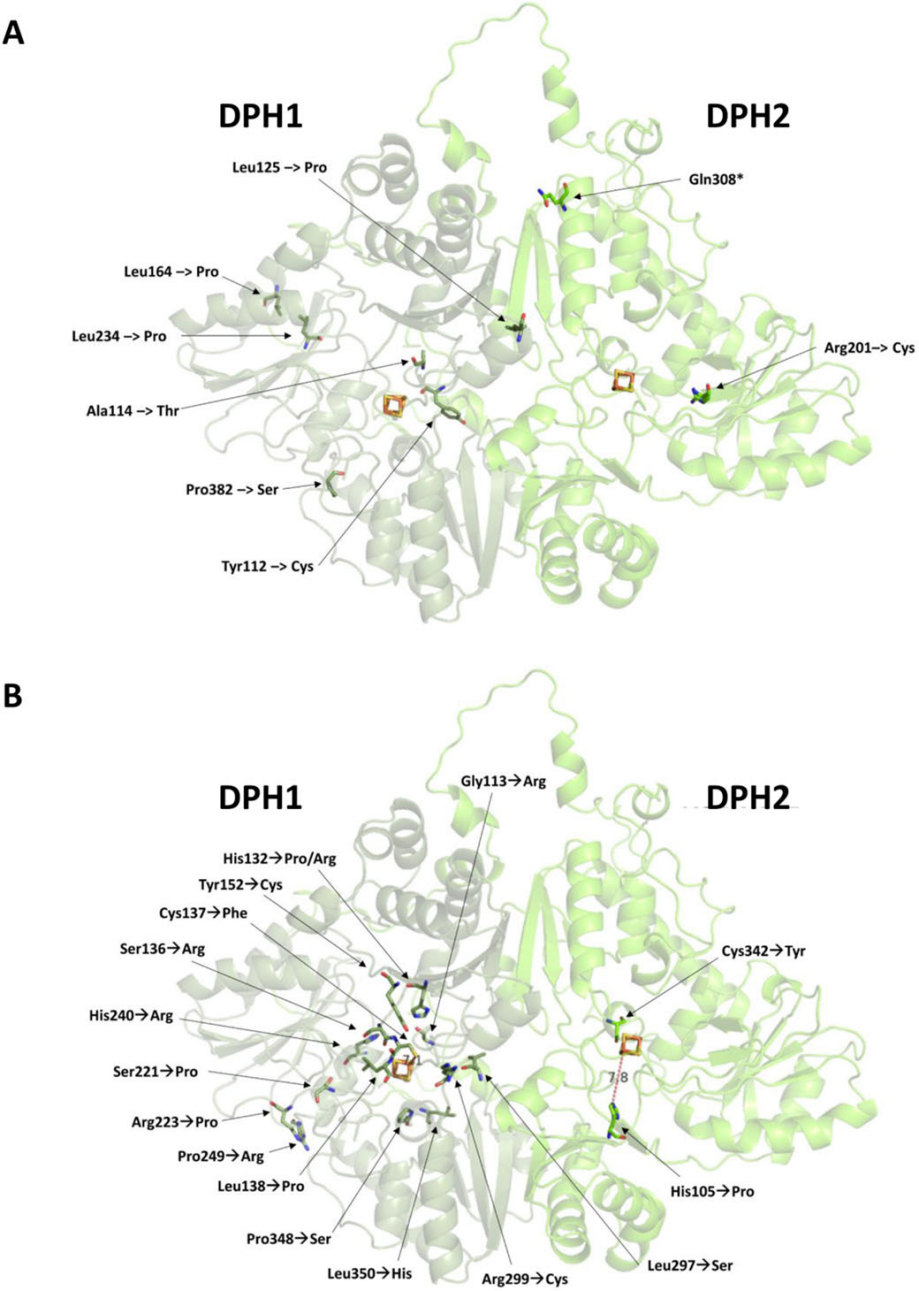
**Structure model of human DPH1/DPH2 and variants.** (A) Variants identified in patients with diphthamide deficiency syndrome and confirmed functionally compromised. (B) Candidate missense variants derived from gnomAD and addressed in this study. Protein structures were modeled via the AlphaFold-based tool ColabFold (Jumper et al., 2021; Mirdita et al., 2022) and illustrated using PyMOL version 1.3.

DPH1 and DPH2 are FeS cluster-containing proteins, which, in concert with DPH3 and DPH4, utilize SAM to modify histidine 715 of human eEF2 with ACP, the first intermediate in diphthamide formation. The diphthamide pathway and enzymes involved are conserved among eukaryotes and archaea, and crystal structures exist for the archaeal homodimer DPH2/DPH2, which is the counterpart of the eukaryotic DPH1/DPH2 heterodimer (Dong et al., 2018; Fenwick et al., 2019; Zhang et al., 2010). Based thereon, structures for human DPH1 and DPH2 and yeast Dph1 and Dph2 can be modeled (Fig. 1), and conserved regions and residues (Dong et al., 2018, 2019) that contribute to FeS cluster and SAM binding and hence are relevant for enzymatic activity can be identified. Fig. 1A and B show the relevant regions and residues in human DPH1/DPH2 and yeast Dph1/Dph2; additional details are provided in Table S2. Based on the definition of functionally relevant regions, positions and residues, the gnomAD database was screened for alleles that encode alterations of DPH1 and DPH2 that might be functionally affected. The mutated residues of interest appeared either in proximity to the active center or potentially impaired their structure owing to mutations from or to a proline residue. The variant alleles that were thereby identified and selected for experimental assessment are listed in Table 1. *DPH1* alleles include G113R, A114T, H132P/R, S136R, C137F, L138P, Y152C, S221P, R223P, H240R, P249R, L297S, R299C, P348S and L350H (Fig. 2). The A114T variant is one of two alleles in the only reported adult patient so far with diphthamide deficiency syndrome (Cheng et al., 2021) (Table S1). In *DPH2*, we analyzed variant alleles that encode H105P and C341Y (Fig. 2). All of the residues are conserved and could therefore unambiguously be aligned to the corresponding yeast enzymes: G131, A132 H150, S154, C155, L156, Y168, S241, R243, H261, P270R, L318, R320, P369 and L371 in yeast Dph1, H123 and C362 in yeast Dph2 (Table 1; Fig. S1). Except for one, all newly identified alleles of interest appear in rare counts (between one and five of 125,748 exomes and 15,708 genomes; *DPH1*, https://gnomad.broadinstitute.org/gene/ENSG00000108963?dataset=gnomad_r2_1; *DPH2*, https://gnomad.broadinstitute.org/gene/ENSG00000132768?dataset=gnomad_r2_1). The positions of these variants in structures of DPH1 and DPH2 are shown in Fig. 2.

**Table 1.**
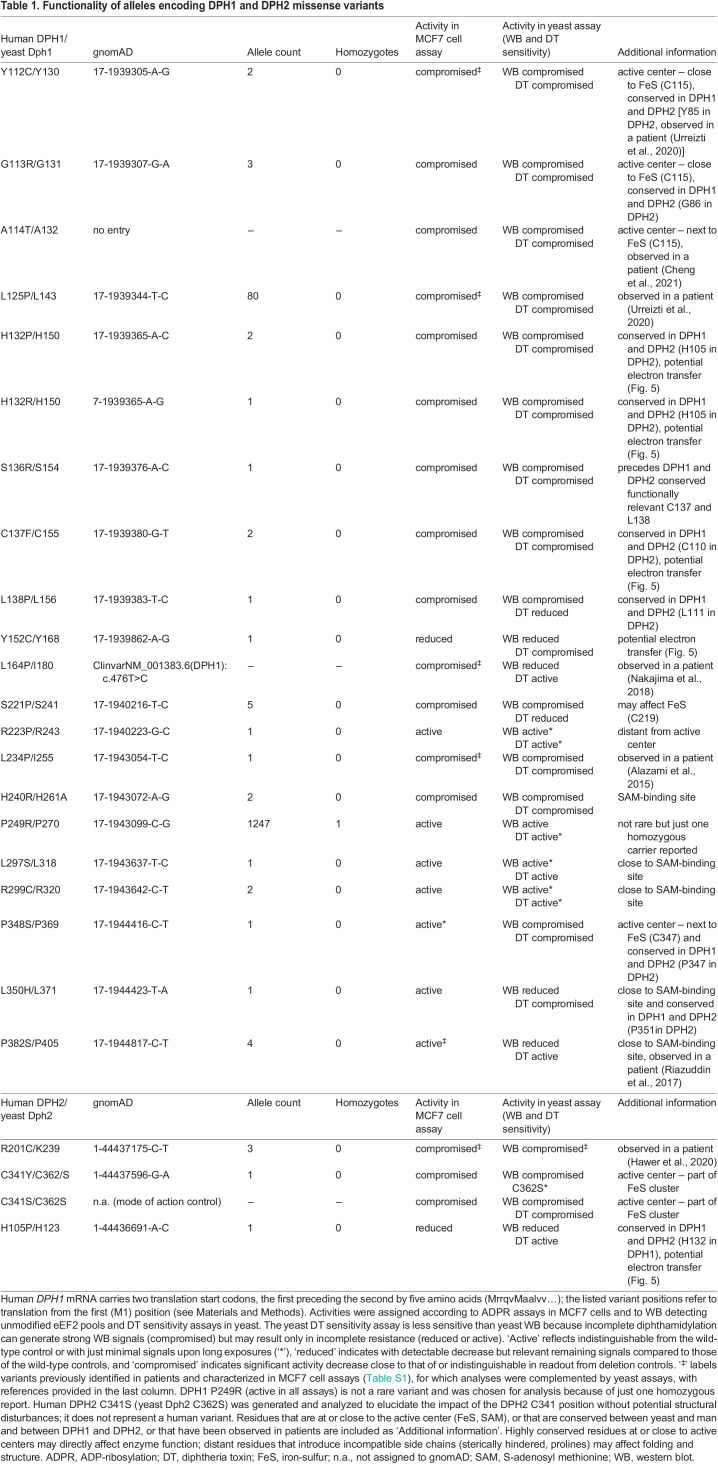
Functionality of alleles encoding DPH1 and DPH2 missense variants

### Functional characterization of DPH1 variants in MCF7 cells and yeast

**Fig. 3.**
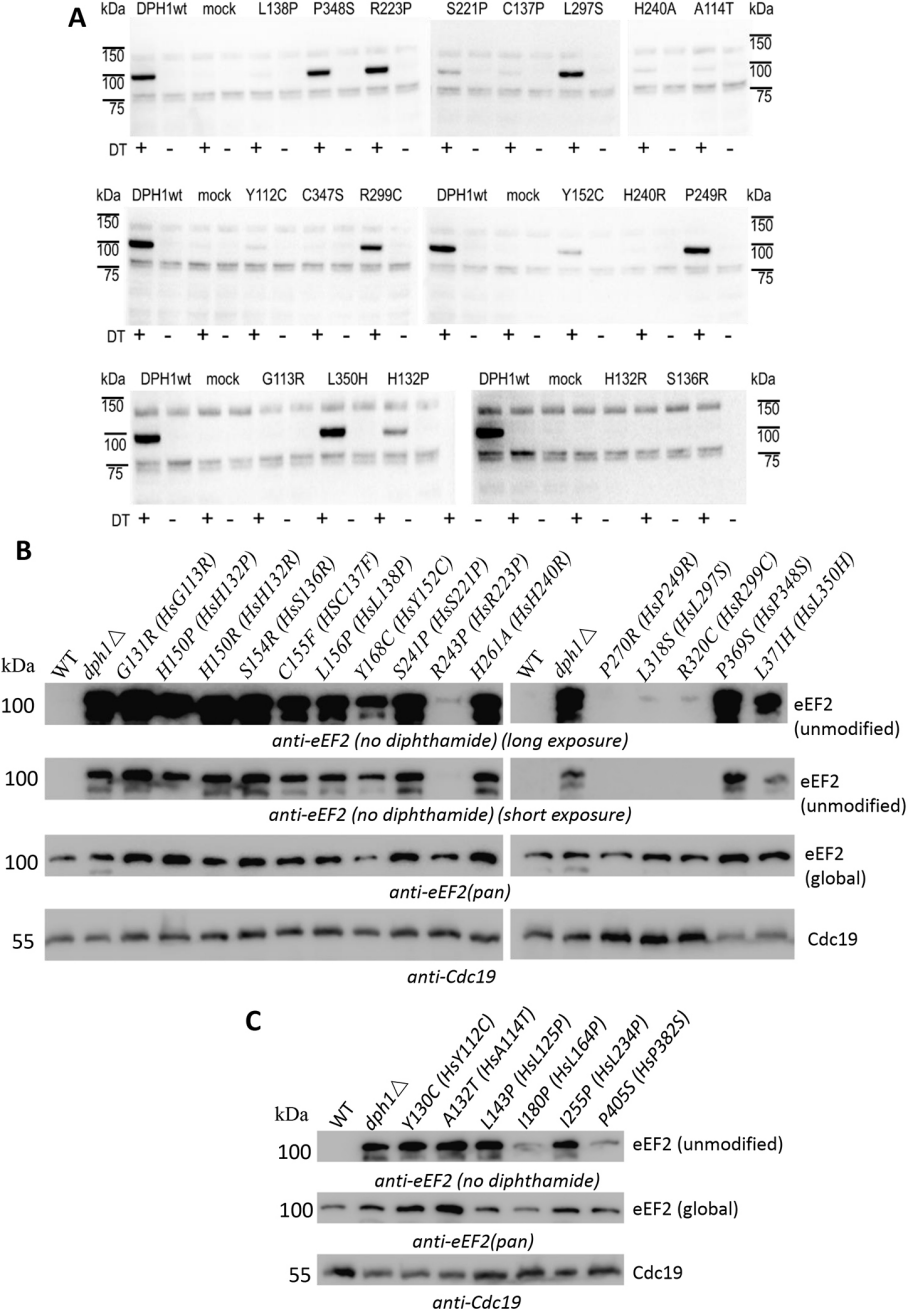
**Functional analyses of DPH1 variants.** (A) ADP-ribosylation (ADPR) assays of human *DPH1*-deficient MCF7 cells that express DPH1 variants. The 100 kDa band visualized by streptavidin-peroxidase reflects eEF2 that carries biotinylated ADP at the diphthamide. ‘+’/‘−’ indicate the presence/absence of diphtheria toxin (DT) in the ADPR assay. wt, wild type. Reduced intensities or absence of 100 kDa signals indicate reduced or absent diphthamide modification. (B,C) Western blot (WB) detection of unmodified eEF2 pools in yeast cells. DPH1 variants display various levels of unmodified eEF2 in comparison to wild-type (WT) and *dph1*Δ controls. WB detection of total eEF2 and yeast pyruvate kinase Cdc19 serve as loading controls.

Rescue of diphthamide synthesis in *DPH1*-deficient human MCF7 cells was applied to assess the functionality of DPH1 enzyme variants encoded by the above rare human alleles. These assays utilize the fact that complementation with wild-type *DPH1* re-enables diphthamide synthesis in DPH1-deficient cells. The presence of diphthamide can be monitored in ADP-ribosylation (ADPR) assays in which exclusively diphthamide-eEF2 becomes modified and can be visualized to indicate the presence of diphthamide on eEF2 (Mayer et al., 2017) (details are provided in the Materials and Methods; expression of variants is shown in Fig. S2B). DPH1 variants, the functions of which are compromised, cannot efficiently rescue diphthamide synthesis and therefore show reduced or lack of signals that represent diphthamide-eEF2. The results of the analyses of the various DPH1 variants in human MCF7 cells are shown in Fig. 3A and summarized in Table 1. Complementation with wild-type *DPH1* showed signals that unambiguously indicated the presence of diphthamide-eEF2. This reflects the activity of the wild-type DPH1 enzyme in our assays. Extracts of cells expressing the variants R223P, P348S, P249R, L297S, R299C and L350H also generated signals close to or at wild-type levels. Thus, these variants (even though affecting conserved positions close to active sites) do not compromise diphthamide synthesis in MCF7 cell assays. In contrast, cells expressing the variants G113R, A114T, H132P, H132R, S136R, C137F, L138P, Y152C, S221P and H240R showed significantly reduced signals. This indicates that the *DPH1* alleles encoding G113R, A114T, H132P, H132R, S136R, C137F, L138P, Y152C, S221P or H240R encode enzymes with compromised functionality.

Functional analyses of these DPH1 variants were also undertaken with mutations introduced at respective positions into the yeast *DPH1* gene from *S. cerevisiae* (details are provided in the Materials and Methods; expression of variants is shown in Fig. S2A). Owing to the high homology of human DPH1 and yeast Dph1, human variants G113R, A114T, H123P/R, S136R, C137F, L138P, Y152C, S221P, R223P, H240R, L297S, R299C, P348S, P249R and L350H could unambiguously be assigned to yeast Dph1 positions G131, A132, H150, S154, C155, L156, Y168, S241, R243, H261, P270, L318, R320, P369 and L371, respectively (Table 1; Fig. S1). The results of these analyses confirmed the functionality assessment of the DPH1 variants in MCF7 cell assays. Fig. 3B shows that diphthamide synthesis [assessed via western blot (WB) analyses specifically detecting pools of unmodified eEF2 in yeast cells] is retained to a large degree in yeast cells. Although a mutation at position P270 resulted in no unmodified eEF2 (fully functional DPH1 variant), very faint signals of unmodified eEF2 were detected in yeast cells expressing R243P, L318S and R320C, indicating that these mutations exert a minor effect on enzyme activity. These mutations in yeast *DPH1* correspond to gnomAD variants of human *DPH1* that retained diphthamide synthesis capabilities in MCF7 cell assays. The yeast P369S and L371H mutants appeared functionally compromised based on detectable levels of unmodified eEF2, although these were not observed in (less sensitive) assays in MCF7 cells expressing the corresponding P348S and L350H variants.

Reduced signals in yeast WB assays were observed for yeast Dph1 mutations G131R, H150P, H150R, S154R, C155F, L156P, Y168C and H261A, which correspond to gnomAD variants of human DPH1 that showed compromised diphthamide synthesis capabilities in MCF7 cell assays. Additionally, human disease relevant variants previously reported and characterized in MCF7 cells (Urreizti et al., 2020) (Table S1) were generated and tested for Dph1 functionality in yeast cells. Human variants Y112C, L125P, L164P, L234P and P382S corresponded to yeast Dph1 residues Y130, L143, I180, I255 and P405 (Table S1). All these clinically relevant alleles showed compromised function, ranging from mild (I180P and P405S) to distinct (Y130C and I255P) loss of activity (Fig. 3C). Yeast Dph1 A132T, corresponding to human DPH1 A114T, also accumulated unmodified eEF2, hence reflected a functional impairment. The yeast variants were also subjected to diphtheria toxin (DT) sensitivity assays *in vivo* (see Materials and Methods) to assess growth defects resulting from inhibition of diphthamide-modified eEF2 by ADPR. DT sensitivity assays are less sensitive than yeast WB because incomplete diphthamide synthesis can generate relevant WB signals but may result only in incomplete DT resistance. The results of these DT sensitivity assays are in line with those observed in WB assays, with gradual differences (compromised versus reduced/active according to WB/DT sensitivity assay, respectively) explainable by the sensitivity differences (Table 1; Fig. S3).

### Functional characterization of DPH2 variants in MCF7 cells and yeast

**Fig. 4.**
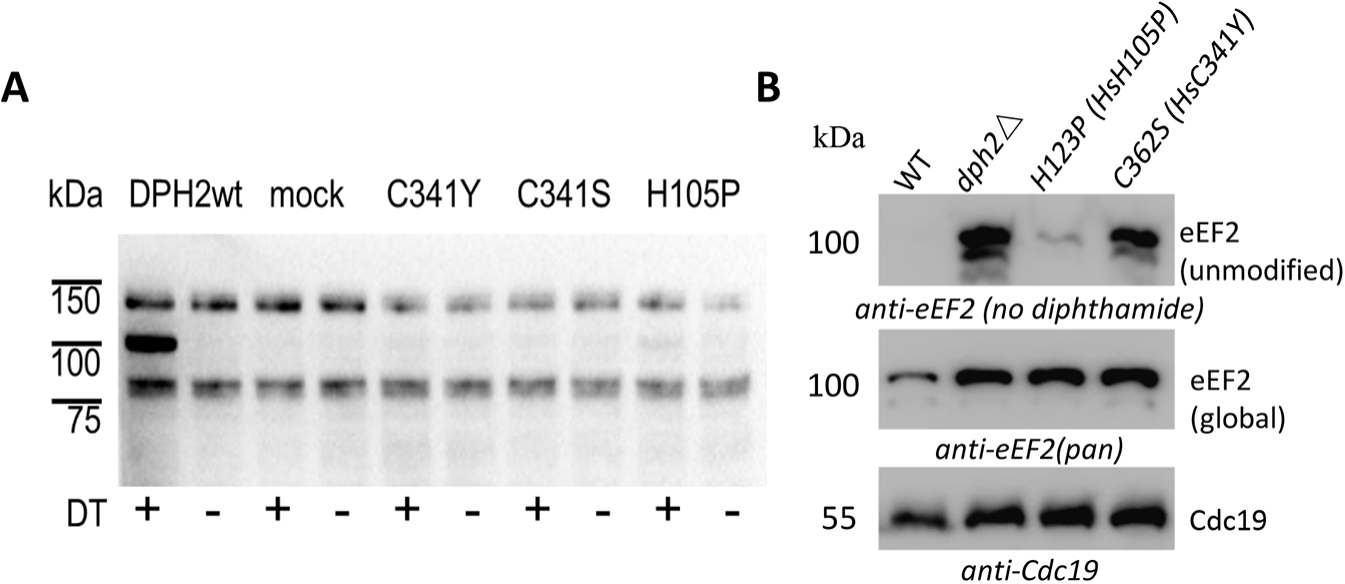
**Functional analyses of DPH2 variants.** (A) ADPR assays of *DPH2-*deficient MCF7 cells. The 100 kDa band visualized by streptavidin-peroxidase reflects eEF2 that carries biotinylated ADP at the diphthamide. ‘+’/‘−’ indicates the presence/absence of DT in the ADPR assay. Reduced intensities or absence of 100 kDa signals indicate reduced or absent diphthamide modification of eEF2. (B) WB detection of unmodified eEF2 in yeast. Dph2 variants display various levels of unmodified eEF2 in comparison to WT and *dph2*Δ controls.

The functionalities of the two so far uncharacterized DPH2 variants H105P and C341Y were analyzed by rescue assays in the same manner as described above for DPH1, with the exception that the capability to compensate diphthamide deficiency was assessed in *DPH2*-deficient MCF7 cells (Fig. 4). Analysis of the H105P variant showed strongly reduced diphthamide synthase activity compared to that of the parent DPH2 enzyme in ADPR assays in MCF7 cells (Fig. 4A). The corresponding WB detection of unmodified eEF2 in yeast (with H123P at the corresponding position in yeast Dph2) confirmed the compromised functionality of this variant, albeit to a lesser degree (Fig. 4B). It is noteworthy that this histidine is conserved not only among human and yeast DPH2, but also between DPH1 and DPH2 (Fig. S1). Interestingly, two human alleles (H132P and H132R) replace that same histidine in DPH1, both also with compromised functionality (Table 1B). The analysis of the C341Y variant in DPH2 revealed a significant reduction in diphthamide synthase activity compared to that of the parent DPH2 enzyme in ADPR assays in MCF7 cells (Fig. 4A). The corresponding eEF2 WB in yeast (with C362S at the corresponding position in Dph2) confirmed the reduced functionality of this variant (Fig. 4B).

## DISCUSSION

Diphthamide deficiency syndrome was first described by Loucks et al. (2015) in children with homozygous *DPH1* variants from consanguineous families and subsequently also reported in compound heterozygous patients (Alazami et al., 2015; Cheng et al., 2021; Nakajima et al., 2018; Riazuddin et al., 2017; Sekiguchi et al., 2018; Urreizti et al., 2020). The syndrome is also associated with variants in other DPH genes, including *DPH2* and *DPH5* (Hawer et al., 2020; Shankar et al., 2022). It is thus not solely defined by loss of function of one single gene but instead by variants in different DPH genes. The number of functionally characterized variants is so far limited to those observed in patients. Genetic variability databases (gnomAD) (Karczewski et al., 2020) reveal additional human *DPH1* and *DPH2* alleles with potentially reduced activity. Their functionality can to some degree be predicted *in silico* with proposed loss of function being reasonable to explain truncations, but unambiguous diagnoses of missense variants need experimental assessment. Because reports on diphthamide deficiency syndrome have raised awareness of the disorder, more patients will likely be identified with so far uncharacterized variants. The herein described characterization of *DPH1* and *DPH2* alleles can support the diagnosis of diphthamide deficiency because variants with reduced functionality are likely to be disease relevant in homozygous or compound heterozygous combinations.

Combined analyses of DPH variants in human and yeast assays provide a more comprehensive assessment of functionality than do individual assays alone. Yeast is evolutionarily distant from human, but its assays based on chromosome-encoded variants can be more sensitive than overexpression-based human cell assays. Conservation of the diphthamide synthesis pathway and essential enzymes compensates the evolutionary distance. Most residues that we analyzed in DPH1 assays are identical in yeast and man (Fig. S1), and so are the key regions of DPH2. In agreement with that, the same results were obtained for most variants in human cell- and yeast-based assays, with few exceptions likely attributable to higher sensitivities of the yeast assays (R223P, L297S, R299C are still wild type-like in MCF7 cells but show some faint signals barely above background in yeast) and only two discrepancies (DPH1 P348S and L350H appear to be tolerated in MCF7 cells, but P369S and L371H reduce activity in yeast). So far, most insights into mechanisms of diphthamide synthesis enzymes including FeS cluster-containing and SAM-binding DPH1 and DPH2 stem from experiments based on *S. cerevisiae* or archaea *Pyrococcus horikoshii* and *Candidatus methanoperedens nitroreducens* (Bär et al., 2008; Dong et al., 2018, 2019; Schaffrath et al., 2014). Our analyses demonstrate that lessons learnt from yeast and archaeal enzymes can be transferred to human counterparts. Variants with reduced activities can be explained by established structure-function interpretations of DPH enzymes or even reveal so far unknown parameters.

Variants that reduce activities explained by established structure-function interpretations include DPH2 C341Y (yeast C362) as part of an FeS cluster-binding domain with functional relevance (Dong et al., 2019). The human variant hence may affect FeS cluster integrity and likely radical SAM functionality. DPH1 G113R, A114T, S221P, Y152C and C137 (all with reduced or compromised functionality) are also in proximity to the active center and hence likely interfere with DPH1 catalysis. Human P348S (yeast P369S) locates next to the FeS cluster at the active center of DPH1, and the yeast assay clearly shows interference with enzyme function in contrast to the less sensitive MCF7 cell assay. Negative interference with enzyme function may also underlie reduced activity of DPH1 H240R and L350H. The yeast counterpart of the latter (L371) was found to be critical for enzyme activity and diphthamide synthesis, presumably through binding of the essential co-factor SAM.

**Fig. 5.**
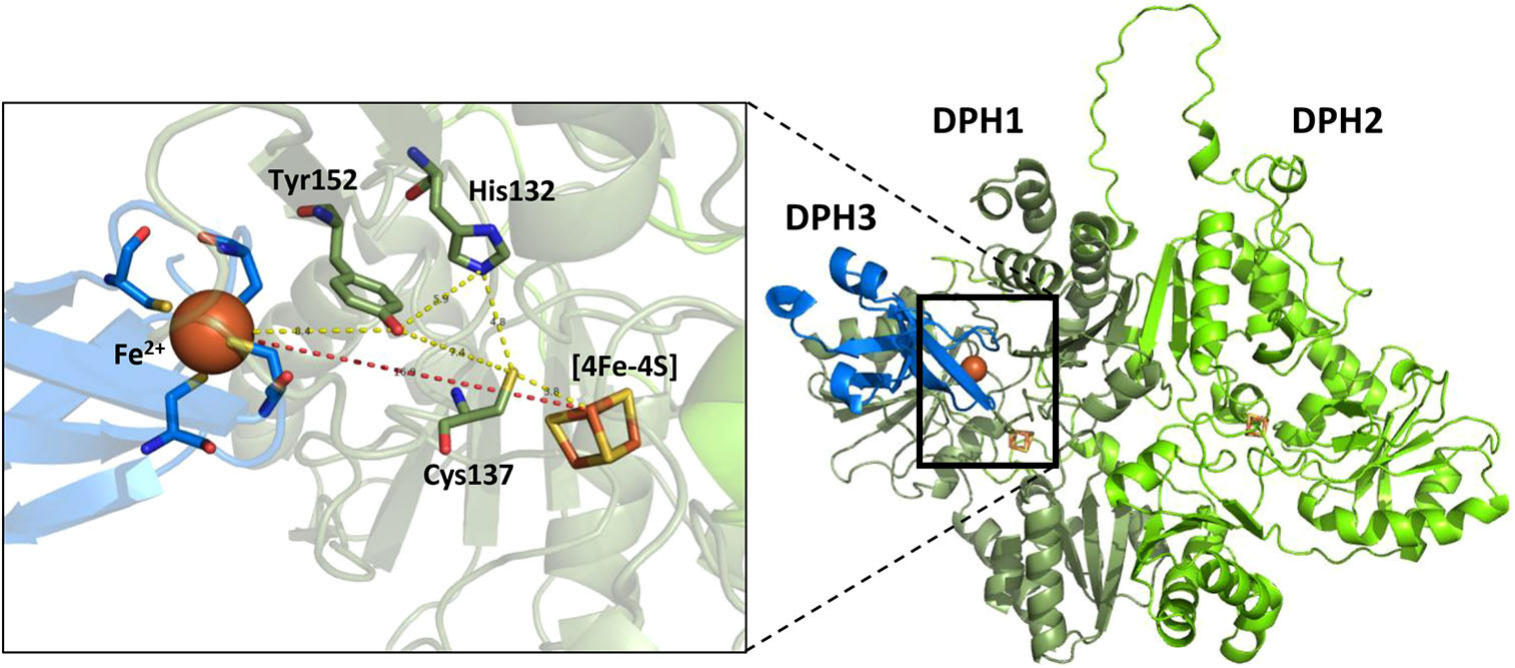
**Structural modeling of human DPH1/DPH2 in interaction with DPH3.** DPH1 residues H132 C137 and Y152 are placed between the DPH3 Fe and DPH1 FeS. These residues may act as electron transfer stepping stones between Fe in DPH3 and DPH1 as an explanation for enzyme deficiencies of the respective mutated alleles. Protein structures were modeled via the AlphaFold-based ColabFold tool (Jumper et al., 2021; Mirdita et al., 2022) and illustrated using PyMOL version 1.3.

Enzyme determinants for which relevance was not previously known were identified by analyzing variants at cross-species and simultaneously cross-enzyme conserved positions. Identical in human and yeast among DPH1 or DPH2, and identical also between DPH1 and DPH2 (Table 1; Fig. S1), were human DPH1 H132 (H105 in DPH2), Y112 (Y85 in DPH2), G113 (G86 in DPH2), C137 (C110 in DPH2) and L138 (L111 in DPH2). All of those variants affect activity. The fact that DPH1 H132P and H132R, and also DPH2 H105P, lose activity suggests that this region is relevant for both enzymes. A model of the DPH1-DPH2 dimer together with DPH3 (Fig. 5) may explain the relevance of several of these positions. DPH3 is the electron donor to reduce the FeS cluster of DPH1 (Dong et al., 2014), but its Fe finger appears too distant for direct electron transfer. It is possible that DPH1 H132, C137 and Y152 (H150, C155 and Y168 in yeast) act as electron transfer stepping stones (Giese et al., 2013) between Fe in DPH3 and DPH1 (Fig. 5). Support for the relevance of this region is also that the compromised S136R and L138P variants (S154 and L156 in yeast) locate right next to the relevant C137 (Fig. 5). Other functionally compromised DPH1 variants previously observed in patients – i.e. L125P, L164P and L234P (Nakajima et al., 2018; Alazami et al., 2015; Urreitzti et al., 2020) – do not locate at or close to the active center. Those variants incorporate (sterically restricted) prolines that may affect protein folding or structure. Not all residues close to the active center or of potential structural relevance affect functionality. DPH1 P249R (yeast P270) is tolerated with full activity in human and yeast assays; R223P (yeast R243), L297S (yeast L318) and R299C (yeast R320S) showed no evidence of activity loss in MCF7 cells and only minuscule amounts of unmodified eEF2 (close to background) in yeast. This underlines the importance of experimental assessment to differentiate deleterious from tolerated variants.

Diphthamide deficiency affects development and manifests with distinct clinical phenotypes in humans, and developmental issues in mice and even plants (Chen and Behringer, 2004; Liu et al., 2006, 2012; Shankar et al., 2022; Zhang et al., 2022). Compromised alleles affect diphthamide synthesis on eEF2 and thus can interfere with translational fidelity and protein homeostasis (Dever et al., 2018; Liu et al., 2012; Shin et al., 2023). The clinical consequences could thus be related to altered translation, resembling ribosomopathies (Farley-Barnes et al., 2019). Translation of selected mRNAs may be sensitive to the presence or absence of diphthamide, such as transcripts with an internal ribosome entry site (IRES) (Argüelles et al., 2014; Godet et al., 2019; Murray et al., 2016; Tsuda-Sakurai and Miura, 2019). A link between diphthamide and IRES-dependent pathway modulation is supported by observations that diphthamide-deficient cells modulate TOR, NF-κB and TNF signaling pathways, which are relevant in development (Mayer et al., 2019; Stahl et al., 2015; Zhang et al., 2022). Elucidation of the genetic and biochemical causes of diphthamide deficiencies can support the diagnosis of diphthamide deficiency syndrome, but treatments do not yet exist. Understanding the enzymes involved, the effects of allelic variants and the biological consequences of reduced diphthamide synthesis may form a basis for better addressing this rare disease in the future.

## MATERIALS AND METHODS

### Definition of potentially compromised human *DPH1* and *DPH2* alleles and of corresponding residues in yeast Dph1 and Dph2 by structure modeling and homology analyses

Structure models of human DPH1 and DPH2 and yeast (*Saccharomyces cerevisiae*) Dph1 and Dph2 proteins were generated by the AlphaFold based tool ColabFold (Jumper et al., 2021; Mirdita et al., 2022) and illustrated in PyMOL version 1.3. For dimer modeling, human DPH1-DPH2 sequences (UniProt Q9BZG8-4 for DPH1 and Q9BQC3-1 for DPH2) were subjected to ColabFold-based dimer modeling. The same procedure was conducted for modeling of the yeast Dph1-Dph2 dimer using UniProt sequences P40487 for Dph1 and P32461 for Dph2. Using PyMOL, generated models were aligned with the Dph2-Dph2 dimer from *Cm. nitroreducens* (PDB: 6BXN) (Dong et al., 2018). Protein structures of 6BXN were hidden, and the [Fe_4_-S_4_] were shown as sticks in representations. Cartoon structures of human DPH1-DPH2 and yeast Dph1-Dph2 were set to 67% transparency to emphasize individual residues of interest shown as sticks. DPH1 and DPH2 are highly conserved between species, and their active centers contain FeS clusters and SAM-binding sites (Dong et al., 2018; Zhang et al., 2010). Potentially compromised human alleles were those that encoded variants close to or at the active center, focusing on changes that might interfere with the enzyme mode of action, those that were observed in patients, or those at critical positions identical between DPH1 and DPH2. Owing to the high homology of the human and yeast enzymes, residues in yeast Dph1 and Dph2 that correspond to the identified human variants were identified by sequence homology analyses (Fig. S1), and their positions were subsequently confirmed in the structure models. Variants in critical positions identical between DPH1 and DPH2 were also selected for analysis. Human *DPH1* mRNA carries two translation start codons, the first preceding the second by five amino acids (MrrqvMaalvv…). The amino acid positions of variants published so far, and for consistency also in this paper, refer to the first start codon, and all variants are unambiguously defined in Fig. S1. Because some reference sequences start at M6, positions in those may need to be (−5) adjusted accordingly.

### Analysis of the activity of DPH1 and DPH2 enzyme variants in human MCF7 cells

To assess the functionality of human DPH1 enzyme variants, pCDNA3-derived plasmids for CMV promoter-driven expression of c-term Flag-tagged cDNA (Mayer et al., 2017) for DPH1 carrying individual variants to be analyzed were transfected into cultured DPH1-deficient human MCF7 breast cancer cells (American Type Culture Collection, ATCC HTB-22). Cell extracts of those transfectants harbor recombinant variant DPH enzymes (Fig. S3) and were subsequently subjected to ADPR assays as previously described (Mayer et al., 2017). In these assays, DT uses biotinylated NAD as a substrate to add biotinylated ADP exclusively to diphthamide-modified eEF2. Biotinylated ADP-diphthamide-eEF2 is visualized on SDS-PAGE protein blots (Bio-Rad, 1704156) with horseradish peroxidase-labeled streptavidin and peroxidase substrate (Roche, 11089153001). Human DPH2 variants were analyzed in the same manner, with expression plasmids for mutated DPH2 transfected into DPH2-deficient human MCF7 cells (Stahl et al., 2015), followed by ADPR assays. These assays base on recombinant overexpression of mutated enzymes and can define inactive and severely compromised enzymes (Mayer et al., 2017). However, compromised enzymes that retain significant residual activities cannot be distinguished from fully active enzymes as overexpression may compensate gradual deficiencies. Transient transfection does not reach all cells, and even transfected cells still retain residual pools of unmodified eEF2 (Fig. S4). Therefore, detection of (reduced/absent) unmodified eEF2 in plasmid-transfected MCF7 cells is (in contrast to chromosome-encoded stable yeast clones) not well suited to assess reconstitution of diphthamide synthesis in our MCF7 cell assay.

### Analysis of the activity of DPH1 and DPH2 enzyme variants in *S. cerevisiae*

The activity of yeast Dph1 and Dph2 variants was analyzed by WB detection of unmodified eEF2 pools in yeast cells, as previously described (Hawer et al., 2018). Therefore, yeast strains expressing Dph1 or Dph2 variants chromosomally under their native promoter were generated by the two-step method (Toulmay and Schneiter, 2006), as previously utilized for generating orthologs of clinically relevant DPH5 variants in yeast cells (Shankar et al., 2022). PCR-mediated pUG73 or pUG27 cassettes (Gueldener, 2002) were integrated into the 3′-untranslated region of *DPH1* or *DPH2*, respectively, in BY4741 cells using standard lithium acetate transformation and selection on minimal yeast nitrogen base (YNB) medium lacking leucine or histidine (Carl Roth, HP26.1). Correct insertion of the pUG73 or pUG27 cassette was verified by diagnostic PCR with primers positioned outside the *DPH1* or *DPH2* locus. Site-directed mutagenesis PCR was performed on a fragment consisting of *DPH1*, including the pUG73 cassette (or *DPH2* including a pUG27 cassette) in the 3′-untranslated region inserted in a pJET1.2 cloning vector (Thermo Fisher Scientific, 1231). The resulting variants were amplified via PCR and transformed in cells via pUG72-based *DPH1* or *DPH2* disruption. After recovery on yeast extract peptone dextrose plates at 30°C overnight, counterselection was performed by growing cells in liquid YNB medium lacking leucine or histidine and containing 1 g/l 5-fluorootic acid (Thermo Fisher Scientific, 10609920) at 30°C for 8 h, and plating 50 μl of the liquid culture on YNB plates lacking leucine or histidine and containing 5-fluorootic acid (1 g/l). Resulting cells expressing variants were sequence verified from their genomic DNA. For extract preparation, strains were grown in full medium overnight, and a fresh culture was inoculated the next day with a start optical density at a wavelength of 600 nm (OD_600_) of 0.2, thereafter harvested at a OD_600_ of 1. To analyze the sensitivity of the generated strains to DT in spot assays (Fig. S3), they were transformed with a galactose-inducible construct expressing the catalytic subunit of DT (pSU9) (Uthman et al., 2013). Tenfold serial dilutions were spotted on minimal YNB medium lacking uracil (Carl Roth, HP26.1) with either 2% glucose or 2% galactose as carbon sources and incubated at 30°C for 3 days. Expression of yeast Dph1 variants (anti-Dph1; Antibodies-online, ABIN2784704) and Dph2 variants [anti-c-myc (9E10); kind gift from Prof. Markus Maniak, University of Kassel, Kassel, Germany] was validated (Fig. S2), detecting Cdc19 (anti-Cdc19; kind gift from Prof. Jeremy Thorner, University of California, Berkeley, Berkeley, CA, USA) as control for sample loading.

## Supplementary Material

10.1242/dmm.050207_sup1 Supplementary information

## First Person

This article has an associated First Person interview with the first author of the paper.
