# Supplementary material for: DPH1 and DPH2 variants that confer susceptibility to diphthamide deficiency syndrome in human cells and yeast models
Source: Dis Model Mech. 2023 Sep 22;16(9):dmm050207. doi: 10.1242/dmm.050207 (PMC10538292; doi:10.1242/dmm.050207)
Supplement: Supplementary information [file dmm-16-050207-s1.pdf]

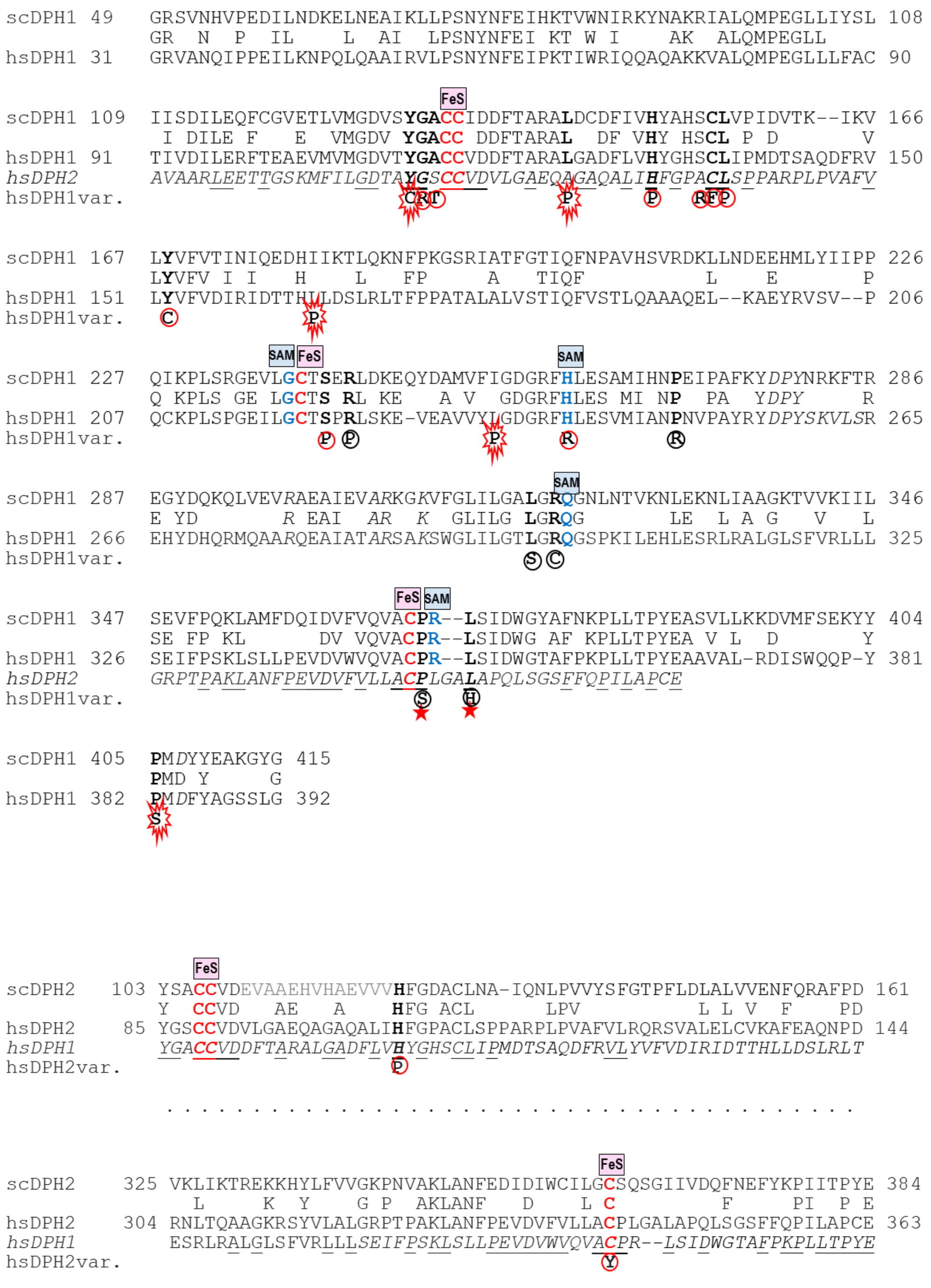

Fig. S1. Alignments of human DPH1 and DPH2 with the enzymes of *S. cerevisiae* show conservation and identities at functionally relevant positions.

FeS and SAM indicate active center residues. Variants previously described in patients are indicated by red asteriscs, black circles indicate database variants for which we did not find functional consequences, red circles are compromised variants. P348 and L350 are conserved among human and yeast DPH1 and also between DPH1 and DPH2. Variants at these positions affect the activity of yeast DPH1 but appear to be tolerated in human enzymes.

Disease Models & Mechanisms • Supplementary information

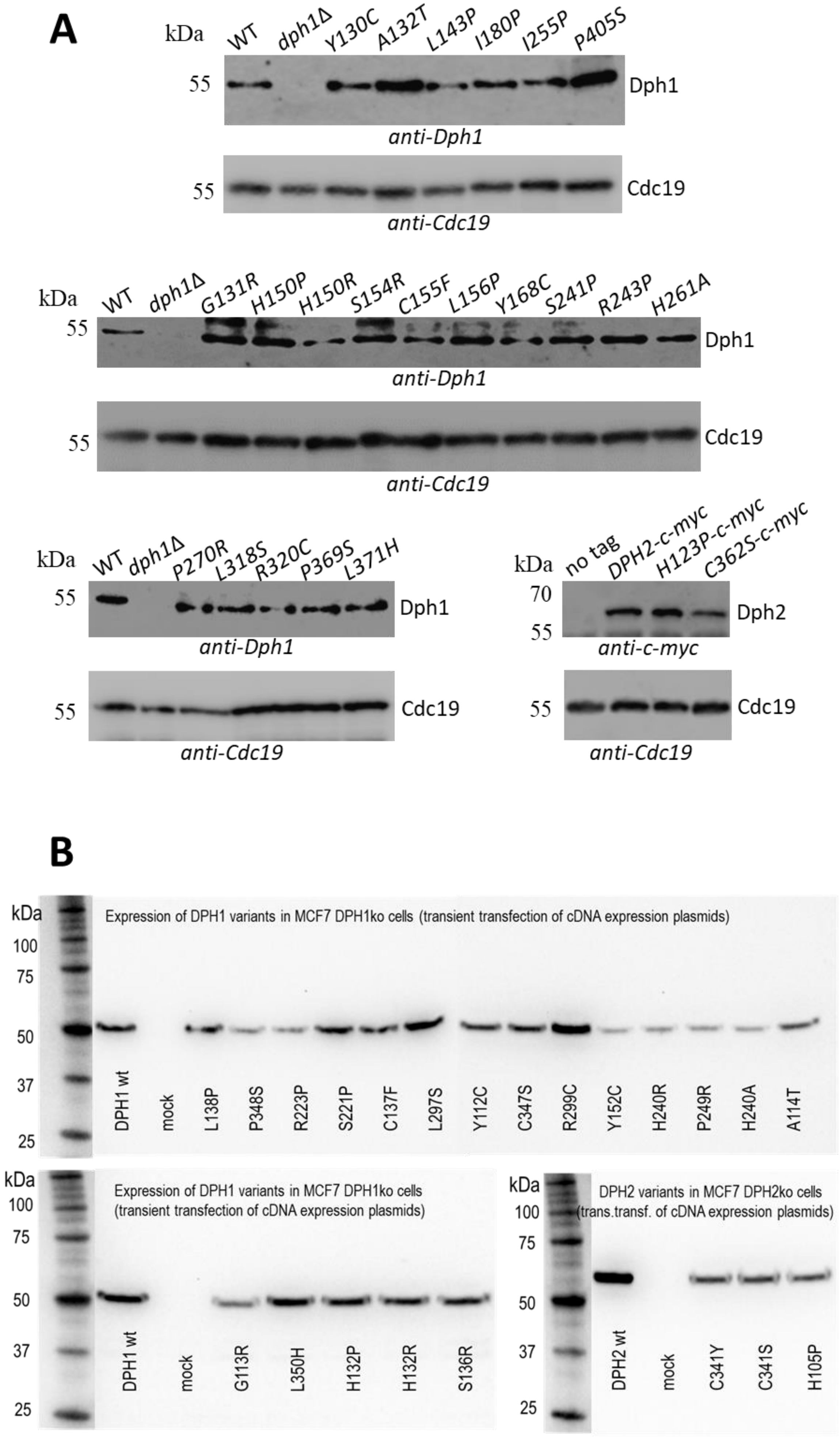

Disease Models & Mechanisms • Supplementary information

Fig. S2. Expression of chromosome-encoded Dph1 and Dph2 variants in yeast and plasmid-encoded DPH1 and DPH2 cDNA in MCF7 cells.

(A) expression in yeast: Total protein extracts of yeast strains expressing Dph1 or DPH2 variants were subjected to Western blot analyses with a polyclonal antibody that detects human and yeast Dph1 (ABIN2784704, Antibodies-Online). Protein extracts of the wild-type and *dph1*Δ served as controls, and yeast pyruvate kinase Cdc19 as independent loading control. lower panel: Yeast strains genomically expressing Dph2 variants harboring C-terminal (c-myc)$_3$ tags were applied to Western blot detection of Dph2 with anti-c-myc antibodies (9E10) according to (Janke et al., 2004). Protein extracts of the wild-type expressing Dph2 with no tag and a strain expressing wild-type Dph2-c-myc, and detection of the yeast pyruvate kinase Cdc19 served as controls.

(B) expression in MCF7: Expression of plasmid-encoded DPH1 cDNA in DPH1ko MCF7 cells and DPH2 cDNA in DPH2ko MCF7 cells. Total protein extracts of DPH1ko MCF7 cells transiently transfected with cDNA encoding DPH1 or -variants were subjected to Western blot analyses with a polyclonal antibody (Sigma-Aldrich cat. no. F7425) that detects the Flag tag added to the C-termini of DPH1. Total protein extracts of DPH2ko MCF7 cells transiently transfected with cDNA encoding DPH2 or -variants were subjected to Western blot analyses with a polyclonal antibody (Sigma-Aldrich cat. no. F7425) that detects the Flag tag added to the C-termini of DPH2.

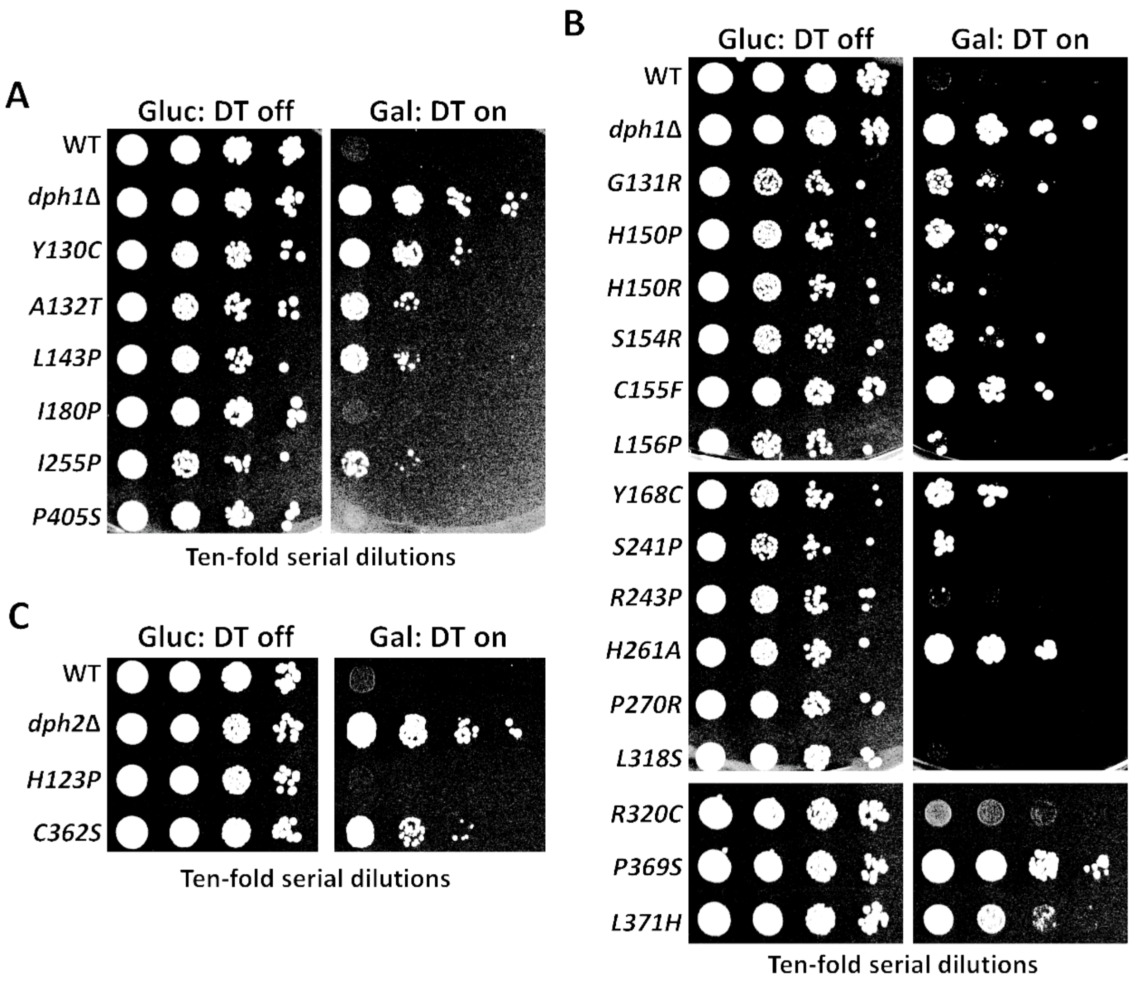

Fig. S3. Sensitivity of yeast strains that express wildtype or variant DPH1 or DPH2 upon exposure to Diphtheria toxin.

Phenotypic spot assay of *DPH1* and *DPH2* strains under exposure towards Diphtheria toxin (DT). A), B) and C): Strains were transformed with a galactose-inducible construct expressing the catalytic subunit of DT (pSU9 according to Uthman et al, (2013)). Ten-fold serial dilutions were spotted on minimal Yeast Nitrogen Base media lacking uracil with either 2% glucose or 2% galactose as carbon sources and incubated at 30°C for three days to visualize survivors in the presence of DT.

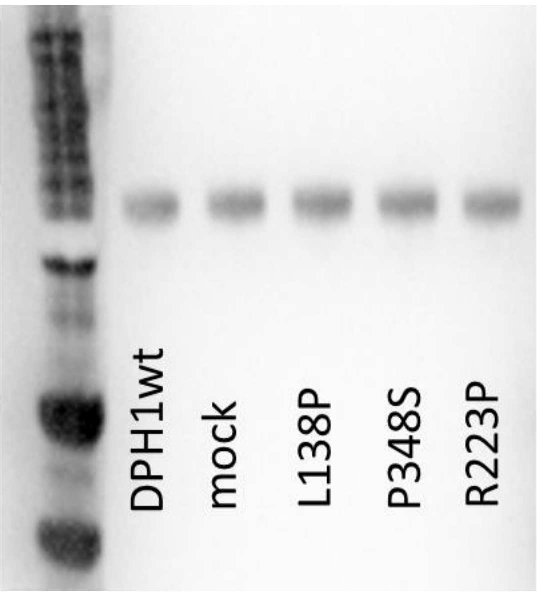

Fig. S4. MCF7-based assays apply ADPR instead of noDiphth antibody to assess enzyme activity because transiently transfected diphthamide deficient MCF7 cell preparations still contain a high background of non-diphthamidylated eEF2.

Unmodified eEF2 was detected in total protein extracts from DPH1ko cells transfected with mock plasmid or DPH1 expression plasmids encoding wt or variant DPH1 protein. Transient transfection does not reach all cells and also transfected cells still retain residual pools of unmodified eEF2. Therefore, Western-blot based assessment of reconstituted diphthamide synthesis with [anti-eEF2(no diphthamide)] is not possible above that background. Therefore, ADPR assays (see Methods, Figures 3 & 4) that base on extracts without ADPR-susceptible diphthamide background were applied to detect enzyme mediated diphthamide signals in MCF7.

Table S1. Mutations of DPH1 and DPH2 in diphthamide deficiency syndrome patients. Human *DPH1* mRNA carries 2 translation start codons, the first preceding the 2nd by 5 amino acids (MrrqvMaalvv…), the listed variant positions refer to translation from the 1st (see methods). Activities were assigned according to ADPR assays in MCF7 and in yeast with western blot (WB) and diphtheria toxin sensitivity (DT) assays, with 'active' being indistinguishable from the WT control or (*) with just minimal signals upon long exposures, 'reduced' with detectable decrease but relevant remaining signals compared to the WT controls, or 'compromised' with significant activity decrease close to or indistinguishable in readout from deletion controls.

HsDPH1 / ScDPH1	clinical phenotype	activity in MCF7 assay	activity in yeast assay	additional information
M6T / n.a.	Loucks et al. 2015	translation initiation cannot be assessed	n.a., yeast TL-initiation region differs	M6 is a conserved start codon in mammals
Y112C / Y130	Urreizti et al.2020	compromised, Urreizti et al. 2020 & this work Table1	compromised, this work Table1	active center - close to FeS(C115)
A114T / A132	Cheng et al. 2021	compromised, this work Table1	compromised, this work Table1	active center - next to FeS(C115)
L125P / L143	Urreizti et al. 2020	compromised, Urreizti et al. 2020	compromised, this work Table1	may affect affect structure or folding
L164P / I180	Nakajima et al. 2018	compromised, Urreizti et al. 2020	reduced, this work Table1	may affect affect structure or folding
L234P / I255	Alazami et al. 2015	compromised, Urreizti et al. 2020	compromised, this work Table1	may affect affect structure or folding
P382S / P405	Riazuddin 2017	active*, Urreizti et al. 2020	reduced, this work Table1	close to SAM-binding site
L96fs*, E97fs* / n.a.	Nakajima et al. 2018	compromised, Mayer et al. 2017	n. a. (frameshift)	truncation
R265Sfs* / n.a.	Cheng et al. 2021	proposed inact., Cheng et al. 2021	n. a. (frameshift)	truncation
A411Rfs* / n.a.	Sekiguchi et al. 2018	compromised, Urreizti et al. 2020	n. a. (frameshift)	truncation
**HsDPH12/ ScDPH2**	**publ. disease**	**MCF7 assay (HsDPH1)**	**yeast assay (ScDPH1)**	**position/effect**
Q308* / K329	Hawer et al. 2020	compromised, Hawer et al. 2020	compromised, Hawer et al. 2020	truncation
R201C / K239	Hawer et al. 2020	compromised, Hawer et al. 2020	compromised, Hawer et al. 2020	may affect affect structure or folding

Table S2. Residues at the active center of DPH1 and DPH2. (A) mutational analyses in yeast define cysteines that contribute to the FeS clusters (Dong et al. 2019). (B) structural analyses of *Cmn*. DPH2/2 proposes residues that are involved in SAM binding (Dong et al., 2018).

**A mutational analyses in yeast (Dong et al. 2019)**			
**ScDPH1**	**HsDPH1**	**position / relevance**	**yeast assays**
C133S	C115	FeS cluster	compromised
C239S	C219	FeS cluster	compromised
C368S	C347	FeS cluster	compromised
**ScDPH2**	**HsDPH2**	**position / relevance**	**yeast assays**
C107S	C89	FeS cluster	compromised
C128S	C110	FeS cluster	compromised
C362S	C341	FeS cluster	compromised

**B Xray structure of Candidatus methanobrevibacter nitroreducens DPH2/2 (Dong et al. 2018)**			
**cmnDPH1/2**	**ScDPH1**	**HsDPH1**	**position / relevance**
G158	G238	G218	SAM binding
H180	H261	H240	SAM binding
Q237	Q321	Q300	SAM binding
V265	V349	I328	SAM binding
R285	R370	R349	SAM binding
D289	D374	D353	SAM binding
D290	W375	W354	SAM binding

Disease Models & Mechanisms • Supplementary information

SUPPLEMENTAL REFERENCES

Alazami, A. M., Patel, N., Shamseldin, H. E., Anazi, S., Al-Dosari, M. S., Alzahrani, F., Hijazi, H., Alshammari, M., Aldahmesh, M. A., Salih, M. A., et al. (2015). Accelerating novel candidate gene discovery in neurogenetic disorders via whole-exome sequencing of prescreened multiplex consanguineous families. *Cell Rep* **10**, 148–161.

Cheng, S. S. W., Luk, H. M. and Lo, I. F. M. (2021). An adult Chinese patient with developmental delay with short stature, dysmorphic features, and sparse hair (Loucks-Innes syndrome). *Am J Med Genet A* **185**, 1925–1931.

Dong, M., Kathiresan, V., Fenwick, M. K., Torelli, A. T., Zhang, Y., Caranto, J. D., Dzikovski, B., Sharma, A., Lancaster, K. M., Freed, J. H., et al. (2018). Organometallic and radical intermediates reveal mechanism of diphthamide biosynthesis. *Science (1979)* **359**, 1247–1250.

Dong, M., Dando, E. E., Kotliar, I., Su, X., Dzikovski, B., Freed, J. H. and Lin, H. (2019). The asymmetric function of Dph1–Dph2 heterodimer in diphthamide biosynthesis. *Journal of Biological Inorganic Chemistry* **24**, 777–782.

Hawer, H., Mendelsohn, B. A., Mayer, K., Kung, A., Malhotra, A., Tuupanen, S., Schleit, J., Brinkmann, U. and Schaffrath, R. (2020). Diphthamide-deficiency syndrome: a novel human developmental disorder and ribosomopathy. *European Journal of Human Genetics* **28**, 1497–1508.

Janke, C., Magiera, M. M., Rathfelder, N., Taxis, C., Reber, S., Maekawa, H., Moreno-Borchart, A., Doenges, G., Schwob, E., Schiebel, E., et al. (2004). A versatile toolbox for PCR-based tagging of yeast genes: New fluorescent proteins, more markers and promoter substitution cassettes. *Yeast* **21**, 947–962.

Loucks, C. M., Parboosingh, J. S., Shaheen, R., Bernier, F. P., Mcleod, D. R., Seidahmed, M. Z., Puffenberger, E. G., Ober, C., Hegele, R. A., Boycott, K. M., et al. (2015). Matching Two Independent Cohorts Validates DPH1 as a Gene Responsible for Autosomal Recessive Intellectual Disability with Short Stature, Craniofacial, and Ectodermal Anomalies. *Hum Mutat* **36**, 1015–1019.

Nakajima, J., Oana, S., Sakaguchi, T., Nakashima, M., Numabe, H., Kawashima, H., Matsumoto, N. and Miyake, N. (2018). Novel compound heterozygous DPH1 mutations in a patient with the unique clinical features of airway obstruction and external genital abnormalities. *J Hum Genet* **63**, 529–532.

Riazuddin, S., Hussain, M., Razzaq, A., Iqbal, Z., Shahzad, M., Polla, D. L., Song, Y., Van Beusekom, E., Khan, A. A., Tomas-Roca, L., et al. (2017). Exome sequencing of Pakistani consanguineous families identifies 30 novel candidate genes for recessive intellectual disability. *Mol Psychiatry* **22**, 1604–1614.

Sekiguchi, F., Nasiri, J., Sedghi, M., Salehi, M., Hosseinzadeh, M., Okamoto, N., Mizuguchi, T., Nakashima, M., Miyatake, S., Takata, A., et al. (2018). A novel homozygous DPH1 mutation causes intellectual disability and unique craniofacial features. *J Hum Genet* **63**, 487–491.

Urreizti, R., Mayer, K., Evrony, G. D., Said, E., Castilla-Vallmanya, L., Cody, N. A. L., Plasencia, G., Gelb, B. D., Grinberg, D., Brinkmann, U., et al. (2020). DPH1 syndrome: two novel variants and structural and functional analyses of seven missense variants identified in syndromic patients. *European Journal of Human Genetics* **28**, 64–75.

Uthman, S., Bär, C., Scheidt, V., Liu, S., ten Have, S., Giorgini, F., Stark, M. J. R. and Schaffrath, R. (2013). The amidation step of diphthamide biosynthesis in yeast requires DPH6, a gene identified through mining the DPH1-DPH5 interaction network. *PLoS Genet* **9**, e1003334.

Disease Models & Mechanisms • Supplementary information
